# Multi-Label Classification in Patient-Doctor Dialogues With the RoBERTa-WWM-ext + CNN (Robustly Optimized Bidirectional Encoder Representations From Transformers Pretraining Approach With Whole Word Masking Extended Combining a Convolutional Neural Network) Model: Named Entity Study

**DOI:** 10.2196/35606

**Published:** 2022-04-21

**Authors:** Yuanyuan Sun, Dongping Gao, Xifeng Shen, Meiting Li, Jiale Nan, Weining Zhang

**Affiliations:** 1 Institute of Medical Information Chinese Academy of Medical Sciences Peking Union Medical College Beijing China; 2 Department of Internal Medicine Chinese Academy of Medical Sciences Peking Union Medical College Hospital Beijing China

**Keywords:** online consultation, named entity, automatic classification, ERNIE, Enhanced Representation through Knowledge Integration, BERT, Bidirectional Encoder Representations from Transformers, machine learning, neural network, model, China, Chinese, classification, patient-physician dialogue, patient doctor dialogue, semantics, natural language processing

## Abstract

**Background:**

With the prevalence of online consultation, many patient-doctor dialogues have accumulated, which, in an authentic language environment, are of significant value to the research and development of intelligent question answering and automated triage in recent natural language processing studies.

**Objective:**

The purpose of this study was to design a front-end task module for the network inquiry of intelligent medical services. Through the study of automatic labeling of real doctor-patient dialogue text on the internet, a method of identifying the negative and positive entities of dialogues with higher accuracy has been explored.

**Methods:**

The data set used for this study was from the Spring Rain Doctor internet online consultation, which was downloaded from the official data set of Alibaba Tianchi Lab. We proposed a composite abutting joint model, which was able to automatically classify the types of clinical finding entities into the following 4 attributes: positive, negative, other, and empty. We adapted a downstream architecture in Chinese Robustly Optimized Bidirectional Encoder Representations from Transformers Pretraining Approach (RoBERTa) with whole word masking (WWM) extended (RoBERTa-WWM-ext) combining a text convolutional neural network (CNN). We used RoBERTa-WWM-ext to express sentence semantics as a text vector and then extracted the local features of the sentence through the CNN, which was our new fusion model. To verify its knowledge learning ability, we chose Enhanced Representation through Knowledge Integration (ERNIE), original Bidirectional Encoder Representations from Transformers (BERT), and Chinese BERT with WWM to perform the same task, and then compared the results. Precision, recall, and macro-F1 were used to evaluate the performance of the methods.

**Results:**

We found that the ERNIE model, which was trained with a large Chinese corpus, had a total score (macro-F1) of 65.78290014, while BERT and BERT-WWM had scores of 53.18247117 and 69.2795315, respectively. Our composite abutting joint model (RoBERTa-WWM-ext + CNN) had a macro-F1 value of 70.55936311, showing that our model outperformed the other models in the task.

**Conclusions:**

The accuracy of the original model can be greatly improved by giving priority to WWM and replacing the word-based mask with unit to classify and label medical entities. Better results can be obtained by effectively optimizing the downstream tasks of the model and the integration of multiple models later on. The study findings contribute to the translation of online consultation information into machine-readable information.

## Introduction

### Background

Internet hospitals in China are in high demand due to limited and unevenly distributed health care resources, lack of family physicians, increasing burden of chronic diseases, and rapid growth of the aging population [1]. Gong et al researched online epidemic-related consultations by multicenter internet hospitals in China during the COVID-19 epidemic, and proved that internet hospitals can offer essential medical support to the public, reduce social panic, and reduce the chance of nosocomial cross-infection, thus playing an important role in preventing and controlling COVID-19 [2]. The COVID-19 outbreak catalyzed the expansion of online health care services. During online consultation, large amounts of text data are accumulated, and contextual data that contain patient-doctor dialogues are of significant value. Network inquiry technology is still in the popularization stage in China, and the text record of inquiry is seldom used in research in the area of natural language processing (NLP), which involves patient privacy and information security [3]. Recently, there has been a lot of work in this area, for instance, a study on the problem of corpus-level entity typing [4]. Chinese scholars have reported on multi-instance learning in the 27th ACM International Conference [5]. Moreover, Wentong et al introduced named entity recognition of electronic medical records based on Bidirectional Encoder Representations from Transformers (BERT) [6] and Piao et al researched a Chinese named entity recognition method based on BERT embedding, which improved entity recognition and attribute labeling [7]. These are significant studies in the NLP domain. Entity studies of clinical text data commonly involve electronic medical records. Dun-Wei et al performed a study based on multi-feature embedding and the attention mechanism [8], and Xue et al researched cross-department chunking [9]. Moreover, Zhang et al studied automatic identification of Chinese clinical entities from free text in electronic health records and contributed to translating human-readable health information into machine-readable information [10]. Furthermore, Jiang et al used machine learning approaches to mine massive service data from the largest China-based online medical consultation platform, which covers 1,582,564 consultation records of patient-physician pairs from 2009 to 2018, and showed that promoting multiple timely responses in patient-provider interactions is essential to encourage payment [11].

However, there is limited clinical dialogue data, and the development of sentence compression for aspect-based sentiment analysis is constantly improving [12]. Chinese researchers have used the BERT model to analyze public emotion during the epidemic of COVID-19 and have substantiated that the fine-tuning of BERT has higher accuracy in the training process [13]. A team from Drexel University used a transformer-based machine learning model to analyze the nuances of vaccine sentiment in Twitter discourse [14]. Patient-doctor dialogues, which are different from daily communication or other universal Q&A, contain important data, such as a patient’s symptoms and the diagnosis by a doctor, and these are called “clinical findings” or named entities in patient-doctor dialogues.

### Objectives

The purpose of this study was to design a front-end task module for the network inquiry of Intelligent Medical Services. Through the study of automatic labeling of real doctor-patient dialogue text on the internet, a method of identifying the negative and positive entities of the dialogue with higher accuracy was explored. This work significantly eliminates the human work involved in feature engineering.

## Methods

### Data Sets

In this paper, our task was named entity automatic classification in patient-doctor dialogues, which was divided into the following 4 attributes: positive, negative, other, and empty. The details are presented below.

The tag “positive (POS)” is used when it can be determined that a patient has dependent symptoms, diseases, and corresponding entities that are likely to cause a certain disease. The tag “negative (NEG)” is used when the disease and symptoms are not related. The tag “other (OTHER)” is used when the user does not know or the answer is unclear/ambiguous, which is difficult to infer. The tag “empty (EMPTY)” is used when there is no practical meaning to determine the patient’s condition, such as interpretation of some medical knowledge by the doctor, independent of the patient’s current condition, inspection items, drug names, etc.

The data set is from the *Spring Rain Doctor* internet online consultation, which has been downloaded from the official data set of Alibaba Tianchi Lab [15]. The training set consists of 6000 dialogues, and each set of dialogues contains more than a dozen statements and a total of 186,305 sentences. The test set consists of 2000 dialogues and a total of 61,207 sentences.

On analysis, we found that online consultation data had the below features.

1. The patient description information was scattered, had slang, and had some spelling mistakes:

患者：经常放屁，很丑(臭) (sentence_id:20); Patient: Fart often. It stnks (stinks)

医生：杀菌治疗的话应该重新换药 (sentence_id:21); Doctor: For bactericidal treatment, you should be replaced with drugs

患者：现在安(按)肚脐左边，感觉按着涨涨的感觉 (sentence_id:22); Patient: Now prress (press) the left side of the navel, I feel it like a balloon.

医生：我觉得这种疼痛应该有中药的影响。(sentence_id:23); Doctor: I think this pain should be affected by traditional Chinese medicine.

2. Interval answers were common:

医生：咳嗽咳痰？(sentence_id:4); Doctor: Any Cough or expectoration?

医生：头痛头晕脑胀？(sentence_id:5); Doctor: Headache, dizziness, or brain swelling?

医生：从资料分析看，有可能是过敏性鼻炎。(sentence_id:6); Doctor: According to the previous examination, it may be allergic rhinitis.

患者：应该是里面，表面上没有鼓包或红肿之类的，没有感冒或咳嗽过最近，头晕脑胀有时会 (sentence_id:7); Patient: It should be inside. There is no bulge or swelling on the surface. There is no cold or cough recently. Dizziness and brain swelling sometimes occur.

3. The main symptoms were mixed with other symptoms:

医生：你好，是10岁的孩子***头痛***吗？(sentence_id:2); Doctor: Hello, is it a 10-year-old child with a *headache*?

患者：是的 (sentence_id:3); Patient: Yes

患者：不知道头疼*恶心吐*，是不是*感冒* (sentence_id:19); Patient: I'm not sure whether headache, *nausea*, or *vomiting* is *colds*

医生：但是感冒一般不会呕吐 (sentence_id:28); Doctor: But a cold usually doesn't cause vomiting

患者：恶心之前*没劲*，*反酸水* (sentence_id:30); Patient: *No strength* before nausea, *sour stomach*

医生：需要详细的问诊和查体，建议到医院*神经内科*或儿童神经内科面诊 (sentence_id:36); Doctor: Need detailed consultation and physical examination, I suggest going to the hospital *neurology department* or children’s neurology department for a face-to-face diagnosis

The above aspects introduce many difficulties in entity recognition and attribute annotation.

The format of raw data was multilayer nested JSON. According to the aspects of the models, we split the innermost text into pairs of splicing contextual sentences. “Jsonlite” is a unique package of R language [16], and the built-in “stream_in” statement does well with tiling JSON into an Excel table, making it intuitive and convenient for us to compare the differences in output data. We then extracted the corresponding subform data according to the analysis requirements. All models shared the same data set. Before input into our model, in addition to the sentence content, we appended the speech role information (ie, sender).

### Composite Abutting Joint Model for Clinical Named Entity Classification

We proposed a composite abutting joint model and adapted a downstream architecture in Chinese Encoder Representations from Transformers Pretraining Approach (RoBERTa) with whole word masking (WWM) extended (RoBERTa-WWM-ext), which combines a text convolutional neural network (CNN) [17]. We used RoBERTa-WWM-ext to express sentence semantics as a text vector [18] and then extracted the local features of the sentence through the CNN, which was our new fusion model.

#### Construction of the Composite Abutting Joint Model

**Figure 1:**
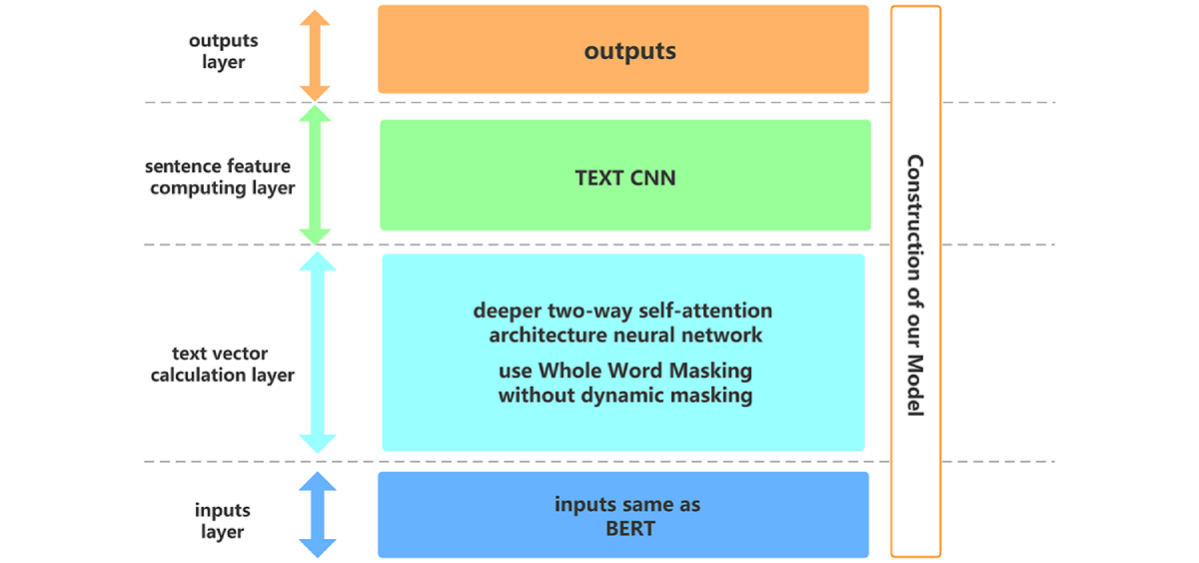
Construction of our model. BERT: Bidirectional Encoder Representations from Transformers; CNN: convolutional neural network.

Chinese RoBERTa-WWM-ext is an open-source model from the Harbin Institute of Technology, which uses WWM combined with the RoBERTa model [19,20]. We adapted a downstream architecture in Chinese RoBERTa-WWM, which combines a text CNN [21]. Our training objective was to use RoBERTa-WWM-ext to express sentence semantics as a text vector and then extract the local features of the sentence through the CNN. The construction of our model is shown in Figure 1.

#### The Input Layer of the Composite Abutting Joint Model

**Figure 2:**
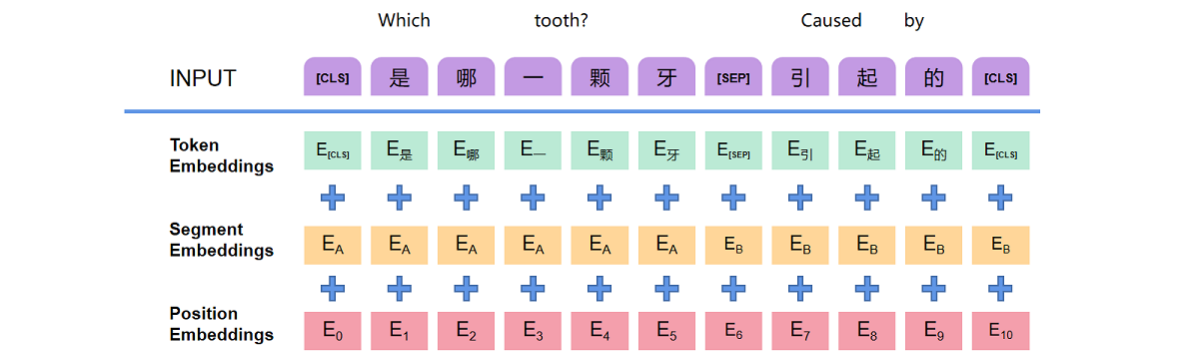
Bidirectional Encoder Representations from Transformers input characterization.

The input layer is the same as BERT [22]. It uses a masked language model (MLM) to generate deep 2-way linguistic representations that combine adjacent and contextual information. Its structure involves stacking traditional transformers, and taking BERT as an example, each of its 12 transformer layers combine left and right contexts to form a deeper 2-way self-attention architecture neural network. Text-input BERT is characterized by 3 levels (Figure 2), namely, token embeddings, segment embeddings, and position embeddings.

#### Text Vector Calculation Layer of the Composite Abutting Joint Model

To maintain continuity between sentences, the beginning and end of the original text are marked with a special symbol [CLS], and the 2 sentences are split with [SEP]. The coded information in the discrete state is transformed into N-dimensional space vectors and transmitted to the encoder unit of the transformer through a continuous and distributed representation. Similarity and distance are computed at the self-attention level to capture word dependencies within sentences. For the calculation of the self-attention function, Vaswani et al introduced “Scaled Dot-Product Attention” [23]. The input includes queries and keys for dimension *d_k_* and the value for dimension *d_v_*. The dot products of a query are computed with all keys, and each is divided by each key. Then, the softmax function is applied to the values. In fact, during the model computation, it has a set of queries packed together into a matrix Q. The keys and values are packed together into matrices K and V. The output matrix is as follows [23]:




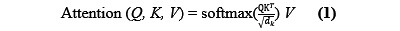




The model could project the queries, keys, and values linearly *h* times with different learned linear projections to *d_k_*, *d_k_*, and *d_v_* dimensions, respectively. On each projected version of the queries, keys, and values, it executes the attention function in parallel to generate *d_v_-*dimensional output values. These values are connected and projected again to obtain the final result. This is multihead attention [23].

Multihead (*Q, K, V*) = Concat (head_1_, ..., head_h_)W^O^
**(2)**

where head_i_ = Attention(*QW_i_^Q^*, *KW_i_^K^*, *VW_i_^V^*) and where the projections are parameter matrices 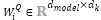, 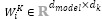, 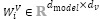, and 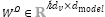.

**Figure 3:**
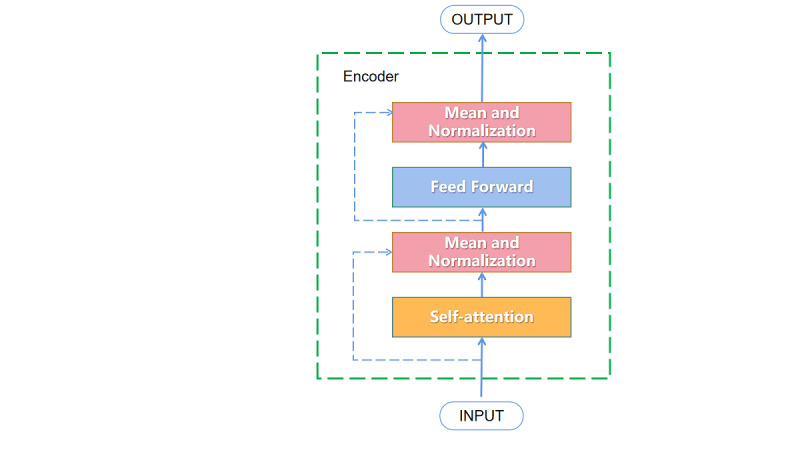
Transformer encoder structure.

The inputs and outputs of the self-attention layer are added and normalized, which makes the output mean of the self-attention layer 0 and the standard deviation 1, and then, it is transferred to the feed-forward layer of the feed-forward neural network. Mean and normalization are processed again. The transformer encoder structure of the model has been described by Vaswani et al [23] (Figure 3).

In transformers, location coding is computed using a trigonometric function as follows [23]:




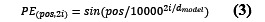







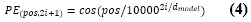




The positional encoding vector results are added to the embedding vector sequence corresponding to each input word instead of connecting vector. Similar to BERT in our model, 15% of the word-piece tokens are masked at random during training. These masked tokens are divided into 3 parts, with 80% of them using [MASK], 10% of them being replaced with a random word, and 10% of them using the original word. Related research by Dandan et al showed that the downstream task of the pretraining model can improve the performance of the model through FINETUNE [24].

During the pretraining phase, the BERT model takes on 2 tasks, MLM and next sentence prediction (NSP). Piao et al have explained the process of predictive masking in MLM tasks, which obtains the semantic representation of a word in a specific context through self-supervised learning [7]. Not the same as BERT, RoBERTa-WWM-ext cancels the NSP and uses max_len = 512 during the pretraining, and the number of training steps is appropriately extended [18].

**Figure 4:**
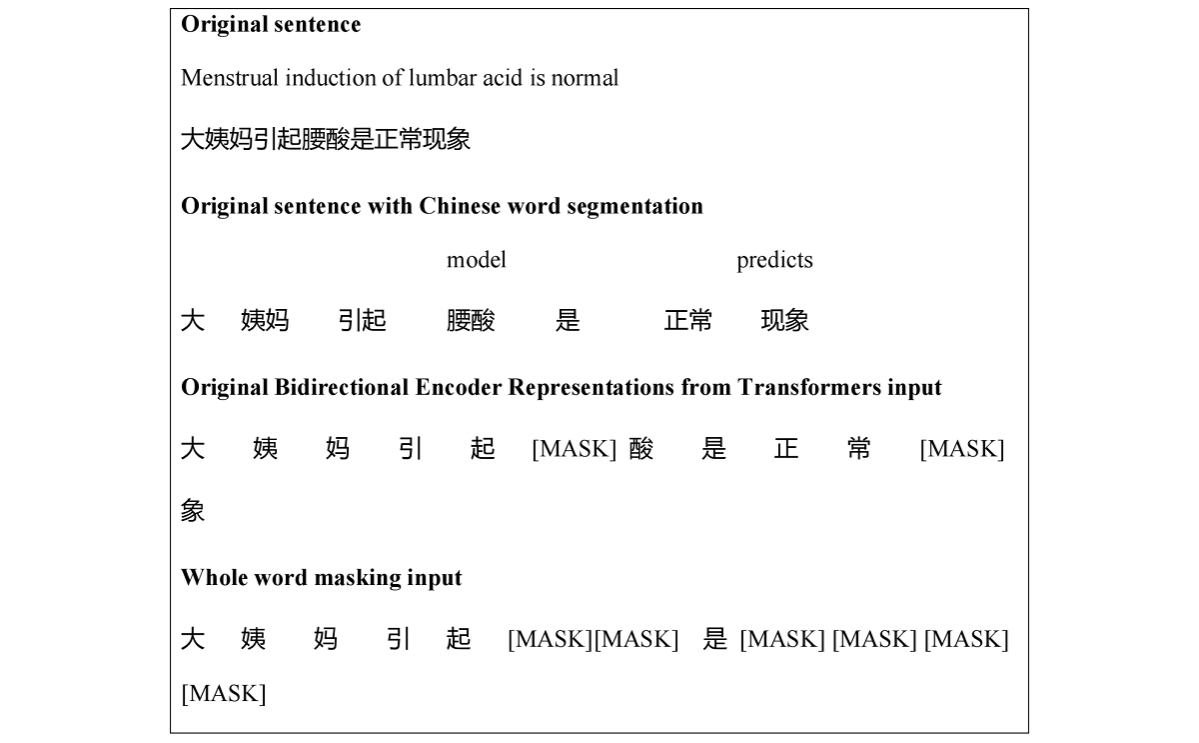
An example of whole word masking in our model.

Another feature of RoBERTa-WWM-ext is that it uses WWM. An example to illustrate the characteristics of WWM is provided in Figure 4 [19].

BERT can only divide Chinese into characters, not words (units). WWM makes the Chinese mask more like English. A complete word will be shielded; otherwise, it will not be shielded, which can maintain the integrity of the Chinese word as a unit, to improve the accuracy of model learning.

#### Sentence Feature Computing Layer of the Composite Abutting Joint Model

The output word vector of RoBERTa-WWM-ext was further extracted by a CNN, which is expected to enhance the robustness of the model. The computing formula is as follows [17,25,26]:




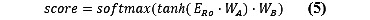







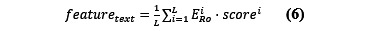







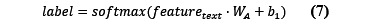




where W_A_ and W_B_ are 2 matrices that are randomly initialized by adding an attention layer to deal with the location characteristics, and b is the RoBERTa-WWM-ext hidden layer dimension, with b_1_ being the offset. Moreover, E_Ro_ represents the output of the coding layer of RoBERTa-WWM-ext, and feature_text_ represents the weighted feature obtained by the product of the score weight and the output of the encoder, which is also the output text vector feature of RoBERTa-WWM-ext. After CNN calculation, the predicted emotion label is finally obtained [27].

## Results

### Evaluation Criteria

We adopted Alibaba cloud’s official evaluation standard, and Macro-F1 was used as the evaluation index. Suppose we have n categories, C1, ..., CI, ..., CN, the calculation is as follows:




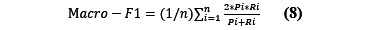




where accuracy (Pi) is the number of samples correctly predicted as category CI/number of samples predicted as category CI, and recall rate (Ri) is the number of samples correctly predicted as category CI/number of samples of the real CI category.

### Graphics Processing Unit Server Requirements

The server requirements are as follows: CPU, 8 cores at 2.5 GHz; memory, 32 GB; hard disk, 500 GB; GPU/Field Programmable Gate Array, 1×NVIDIA V100.

### Results of Our Composite Abutting Joint Model

Our data involved a 3-layer nested JSON file. The first layer was regarded as the index of each dialogue, the second layer was the specific dialogue content between patients and doctors in each dialogue, and the third layer was the entity part corresponding to a single sentence. Not every sentence had an entity part, and not every entity needed to be marked with an entity attribute. We expanded the training set data and all the models’ training results. The distribution of entity attribute labels is shown in Table 1.

From Table 1, we know that the BERT results of the test data have more positive labels, with a value nearly 10 percentage points higher than that for the train data, and the negative labels were nearly 4 percentage points less than that for the train data. After optimizing WWM, the attribute proportion was close to the train data, but there was still a certain gap. We used the fine-tune approach with CNN for RoBERTa-WWM-ext, but it did not change the label proportion. In the Enhanced Representation through Knowledge Integration (ERNIE) model train results, the attribute proportion was closer to that for the train data when compared with BERT. Next, we compared the 4 models, and the results are shown in Table 2.

**Table 1:** Attribute statistics in the training data and the model training results of the test data.

Data set	Training data (N=118,976)	Test data (N=39,204)			
		ERNIE^a^	BERT^b^	BERT-WWM^c^	RoBERTa-WWM-ext + CNN^d^
POS^e^, n (%)	74,774 (62.85%)	25,163 (64.18%)	27,866 (71.08%)	26,116 (66.62%)	26,116 (66.62%)
NEG^f^, n (%)	14,086 (11.84%)	4271 (10.89%)	3125 (7.97%)	3871 (9.87%)	3871 (9.87%)
OTHER^g^, n (%)	6167 (5.18%)	1006 (2.57%)	684 (1.74%)	2587 (6.60%)	2587 (6.60%)
EMPTY^h^, n (%)	23,949 (20.13%)	8764 (22.35%)	7529 (19.20%)	6630 (16.91%)	6630 (16.91%)

^a^ERNIE: Enhanced Representation through Knowledge Integration.

^b^BERT: Bidirectional Encoder Representations from Transformers.

^c^BERT-WWM: Bidirectional Encoder Representations from Transformers with whole word masking.

^d^RoBERTa-WWM-ext + CNN: Robustly Optimized BERT Pretraining Approach with whole word masking extended plus a convolutional neural network.

^e^The tag “positive (POS)” is used when it can be determined that a patient has dependent symptoms, diseases, and corresponding entities that are likely to cause a certain disease.

^f^NEG: The tag “negative (NEG)” is used when the disease and symptoms are not related.

^g^OTHER: The tag “other (OTHER)” is used when the user does not know or the answer is unclear/ambiguous, which is difficult to infer.

^h^EMPTY: The tag “empty (EMPTY)” is used when there is no practical meaning to determine the patient’s condition, such as interpretation of some medical knowledge by the doctor, independent of the patient’s current condition, inspection items, drug names, etc.

**Table 2:** The scores of the 4 models.

Data set	ERNIE^a^	BERT^b^		
		BERT	BERT-WWM^c^	RoBERTa-WWM-ext + CNN^d^
POS^e^-Rr^f^	87.32461545	87.10998052	89.81676537	89.23248142
POS-Pr	87.35933834	78.69582391	86.57854406	88.20871479
POS-F1	87.34197344	82.68940537	88.16793149	88.71764473
NEG^g^-Rr^h^	67.70158588	41.50100514	66.96448515	70.13625195
NEG-Pr	71.03351301	59.45600000	77.50775595	77.30182176
NEG-F1	69.32753888	48.88187319	71.85140803	73.54491158
OTHER^i^-Rr	27.30551262	12.98299845	58.06285420	57.13549717
OTHER-Pr	52.68389662	36.84210526	43.58081980	45.06298253
OTHER-F1	35.96878181	19.20000000	49.79014800	50.38618810
EMPTY^j^-Rr	75.84846093	61.62851881	62.98342541	67.77163904
EMPTY-Pr	65.84446728	62.29224837	72.27169811	71.50589868
EMPTY-F1	70.49330644	61.95860610	67.30863850	69.58870804
Macro-Rr	64.54504372	50.80562573	69.45688253	71.06896740
Macro-Pr	69.23030381	59.32154439	69.98470448	70.51985444
Total score (Macro-F1)	65.78290014	53.18247117	69.27953150	70.55936311

^a^ERNIE: Enhanced Representation through Knowledge Integration.

^b^BERT: Bidirectional Encoder Representations from Transformers.

^c^BERT-WWM: Bidirectional Encoder Representations from Transformers with whole word masking.

^d^RoBERTa-WWM-ext + CNN: Robustly Optimized BERT Pretraining Approach with whole word masking extended plus a convolutional neural network.

^e^The tag “positive (POS)” is used when it can be determined that a patient has dependent symptoms, diseases, and corresponding entities that are likely to cause a certain disease.

^f^Pr: precision rate.

^g^NEG: The tag “negative (NEG)” is used when the disease and symptoms are not related.

^h^Rr: recall rate.

^i^OTHER: The tag “other (OTHER)” is used when the user does not know or the answer is unclear/ambiguous, which is difficult to infer.

^j^EMPTY: The tag “empty (EMPTY)” is used when there is no practical meaning to determine the patient’s condition, such as interpretation of some medical knowledge by the doctor, independent of the patient’s current condition, inspection items, drug names, etc.

## Discussion

From the scoring results, the ERNIE model, which has been trained on a large Chinese corpus, had a total score 12.6 points higher than that of the BERT model in our task. BERT-WWM surpassed ERNIE, with a score of 69.28. Our RoBERTa-WWM-ext + CNN model improved the overall score by 1.28. With the addition of the message sender in the corpus of RoBERTa-WWM-ext, the correct rate of answering sentences also improved.

A previous report assessed BERT fine-tuning as embedding input into the text CNN model and showed that the accuracy rate was 0.31% higher than that of the original BERT model and was more stable [28]. We used CNN to compute sentence features. To verify our model’s knowledge learning ability, we chose ERNIE [29], original BERT, and Chinese BERT with WWM to do the same task, and then compared the results of these models.

In this study, we showed that our model outperformed the other models on the task. The test was not manually modified, and the error of the training data limited the role of manual rules. We tried to add rules to correct the positive labeling, but the total score was only 29.31 points. The accuracy of the positive label was 92.33, but the recall was only 16.46. Due to the false-positive interference of the original data, it was difficult to improve the accuracy of the model itself through artificial rules. The longest sequence length supported by BERT is 512. The text tasks suitable for processing include short texts, such as comments on social platforms and article titles, but for a medical dialogue composed of more than 50 single sentences, the length is obviously not enough. We can only use the truncation method to preprocess text, that is, first truncation, tail truncation, and head to tail truncation, which adds some difficulty to the preliminary work. According to the work of Zeng et al, the base model did improve the accuracy rate by adjusting the downstream tasks [30]. For the single model, XLNET and RoBERTa were better than BERT and ERNIE, and the integration of multiple models will improve the model by 2.58% on average. The results of this study indicated that the accuracy of the model improved with small and middle sample sizes. The multimodel joint integration was an effective way to improve the accuracy of the entity attribute annotation.

“Internet medical+” was part of China’s rapid development after “Internet+” became China’s national strategy in 2015 [31]. In 2019, the novel coronavirus pneumonia outbreak occurred globally, and traditional medical treatment brought many malpractices, which stimulated the technical development of internet inquiry [32]. In the 9th IEEE International Conference on Health Care Informatics (ICHI) in 2021, some scholars proposed to integrate structured data with unstructured text annotation recorded in the classification stage, and use NLP methods for admission prediction and triage notes [33]. This study hopes to further optimize medical information and pave the way for the automatic generation of medical cases through the automatic entity annotation of doctor-patient real dialogue text generated in the process of consultation. It is speculated that our study findings will contribute to the application of NLP methods in the field of health care.

## Abbreviations

BERT: Bidirectional Encoder Representations from Transformers
CNN: convolutional neural network
ERNIE: Enhanced Representation through Knowledge Integration
MLM: masked language model
NLP: natural language processing
NSP: next sentence prediction
RoBERTa: Robustly Optimized Bidirectional Encoder Representations from Transformers Pretraining Approach
RoBERTa-WWM-ext: Robustly Optimized Bidirectional Encoder Representations from Transformers Pretraining Approach with whole word masking extended
WWM: whole word masking

## References

[1] Jiang X, Xie H, Tang R, Du Y, Li T, Gao J, Xu X, Jiang S, Zhao T, Zhao W, Sun X, Hu G, Wu D, Xie G (2021). Characteristics of Online Health Care Services From China's Largest Online Medical Platform: Cross-sectional Survey Study. J Med Internet Res.

[2] Gong K, Xu Z, Cai Z, Chen Y, Wang Z (2020). Internet Hospitals Help Prevent and Control the Epidemic of COVID-19 in China: Multicenter User Profiling Study. J Med Internet Res.

[3] Dang Y, Guo S, Guo X, Vogel D (2020). Privacy Protection in Online Health Communities: Natural Experimental Empirical Study. J Med Internet Res.

[4] Yaghoobzadeh Y, Adel H, Schuetze H (2018). Corpus-Level Fine-Grained Entity Typing. Journal of Artificial Intelligence.

[5] Xu B, Luo Z, Huang L, Liang B, Xiao Y, Yang D, Wang W (2018). METIC: Multi-Instance Entity Typing from Corpus. CIKM '18: Proceedings of the 27th ACM International Conference on Information and Knowledge Management.

[6] Wentong L, Yanhui Z, Fei Z, Xiangbing J (2020). Named Entity Recognition of Electronic Medical Records Based on BERT. Journal of Hunan University of Technology.

[7] Piao Y, Wenyong D (2020). Chinese Named Entity Recognition Method Based on BERT Embedding. Computer Engineering.

[8] Dun-Wei G, Yong-Kai Z, Yi-Nan G, Bin W, Kuan-Lu F, Yan H (2021). Named entity recognition of Chinese electronic medical records based on multifeature embedding and attention mechanism. Chinese Journal of Engineering.

[9] Xue D, Zhipeng J, Yi G (2017). Cross-department chunking based on Chinese electronic medical record. Application Research of Computers.

[10] Zhang Y, Wang X, Hou Z, Li J (2018). Clinical Named Entity Recognition From Chinese Electronic Health Records via Machine Learning Methods. JMIR Med Inform.

[11] Jiang J, Cameron A, Yang M (2020). Analysis of Massive Online Medical Consultation Service Data to Understand Physicians' Economic Return: Observational Data Mining Study. JMIR Med Inform.

[12] Che W, Zhao Y, Guo H, Su Z, Liu T (2015). Sentence Compression for Aspect-Based Sentiment Analysis. IEEE/ACM Trans. Audio Speech Lang. Process.

[13] Wang T, Lu K, Chow KP, Zhu Q (2020). COVID-19 Sensing: Negative Sentiment Analysis on Social Media in China via BERT Model. IEEE Access.

[14] Kummervold PE, Martin S, Dada S, Kilich E, Denny C, Paterson P, Larson HJ (2021). Categorizing Vaccine Confidence With a Transformer-Based Machine Learning Model: Analysis of Nuances of Vaccine Sentiment in Twitter Discourse. JMIR Med Inform.

[15] (2021). CBLUE: A Chinese Biomedical Language Understanding Evaluation Benchmark. Alibaba Group.

[16] Ihaka R, Gentleman R (1996). R: A Language for Data Analysis and Graphics. Journal of Computational and Graphical Statistics.

[17] Pattanayak S (2017). Convolutional Neural Networks. Pro Deep Learning with TensorFlow.

[18] Liu Y, Ott M, Goyal N, Du J, Joshi M, Chen D, Levy O, Lewis M, Zettlemoyer L, Stoyanov V (2019). RoBERTa: A Robustly Optimized BERT Pretraining Approach. arXiv.

[19] Cui Y, Che W, Liu T, Qin B, Yang Z (2021). Pre-Training With Whole Word Masking for Chinese BERT. IEEE/ACM Trans. Audio Speech Lang. Process.

[20] Wolf T, Debut L, Sanh V, Chaumond J, Delangue C, Moi A, Cistac P, Rault T (2020). HuggingFace's Transformers: State-of-the-art Natural Language Processing. arXiv.

[21] Lei T, Barzilay R, Jaakkola T (2015). Molding CNNs for text: non-linear, non-consecutive convolutions. arXiv.

[22] Devlin J, Chang M, Lee K, Toutanova K (2018). BERT: Pre-training of Deep Bidirectional Transformers for Language Understanding. arXiv.

[23] Vaswani A, Shazeer N, Parmar N, Uszkoreit J, Jones L, Gomez A, Kaiser L, Polosukhin I (2017). Attention Is All You Need. arXiv.

[24] Dandan D, Jiashan T, Yong W, Kehai Y, Jiashan T, Yong W (2021). Chinese Short Text Classification Algorithm Based on BERT Model. Computer Engineering.

[25] Dauphin Y, Fan A, Auli M, Grangier D (2017). Language Modeling with Gated Convolutional Networks. arXiv.

[26] Chen Z, Qian T (2019). Transfer Capsule Network for Aspect Level Sentiment Classification. Proceedings of the 57th Annual Meeting of the Association for Computational Linguistics.

[27] Kun W, Yi Z, Shuya F, Shouyin L (2020). Long text aspect-level sentiment analysis based on text filtering and improved BERT. Journal of Computer Applications.

[28] Xiaowei Z, Jianfei S (2021). Research on News Text Classification Based on Improved BERT-CNN Model. Video Engineering.

[29] Sun Y, Wang S, Li Y, Feng S, Chen X, Zhang H, Tian X (2019). ERNIE: Enhanced Representation through Knowledge Integration. arXiv.

[30] Zeng K, Xu Y, Lin G, Liang L, Hao T (2021). Automated classification of clinical trial eligibility criteria text based on ensemble learning and metric learning. BMC Med Inform Decis Mak.

[31] Xiaoyan Z, Jing B, Rong L (2019). Studying on The Existing Modes of “Internet Plus Medical Services” in China. Chinese Health Service Management.

[32] Hui C, Qiong Z, Xiaoli L, Bochun Y (2020). Opportunity and Reflection of the lnternet+Medical Under COVlD-19 Epidemic Situation. Chinese Hospital Management.

[33] Arnaud É, Elbattah M, Gignon M, Dequen G (2021). NLP-Based Prediction of Medical Specialties at Hospital Admission Using Triage Notes.

